# Assessing mental resilience with individual and lifestyle determinants among nursing students: An observational study from Greece

**DOI:** 10.3934/publichealth.2024049

**Published:** 2024-08-21

**Authors:** Maria Antoniou, Evangelos C. Fradelos, Theano Roumeliotaki, Foteini Malli, Emmanouil, K. Symvoulakis, Dimitrios Papagiannis

**Affiliations:** 1 Public Health & Adults immunization Laboratory, Faculty of Nursing, University of Thessaly, 41500, Larissa, Greece; 2 Laboratory of Clinical Nursing, Department of Nursing, University of Thessaly, 41500 Larissa, Greece; 3 Clinic of Preventive Medicine and Nutrition, Department of Social Medicine, Medical School, University of Crete, Heraklion, Greece; 4 Respiratory Disorders Lab, Faculty of Nursing, University of Thessaly, 41500 Larissa, Greece; 5 Department of Social Medicine, Medical School, University of Crete, Heraklion, Greece

**Keywords:** mental resilience, perceived stress, trust and connections, nursing students, education

## Abstract

The educational environment is important for the development of life skills of nursing students in late- and post-adolescence. Strengthening their mental resilience, enhancing their individual confidence, and controlling stress are necessary conditions in this direction, which will help them cope with the future challenges of their chosen profession. We aimed to study the resilience profiles of nursing students by investigating their mental resilience and its association with their individual characteristics and lifestyle factors. The Connor-Davidson Resilience Scale (CD–RISC–25) scale for resilience, the Perceived Stress Scale (PSS–14) scale for perceived stress, and the Personal Trust and Connection (PerTC) scale for trust and connections were used. The lifestyle determinants were also assessed. An e-survey that targeted 250 nursing students was conducted from November 22 to April 23. Descriptive and advanced statistical analyses were performed. 146 students participated in the study via an on-line questionnaire; the students were predominantly female (82.2%) with mean age of 22 years (*SD* = 6.8). Two out of ten students smoked (20.5%), 66.4% consumed at least one drink during a usual week, and 48.0% participated in sports during the last year. The mean hours spent on the Internet daily was 4.2 (*SD* = 1.8) and on social media was 2.7 (*SD* = 1.6) hours/day. The students scored highly on the 1-to-10 life-satisfaction item (*Mean* = 6.3, *SD* = 1.9), where the perceived stress was assessed as moderate/high with a mean of 33 (*SD* = 4.4) and trust and connections had a mean of 6.2 (*SD* = 1.1). In the multivariate analysis, the factors found to significantly associate with resilience (CD–RISC–25; *Mean* = 64.2, *SD* = 11.8) were age (*β* = 0.4; 95% *CI*: 0.1, 0.7), sports participation (*β* = 5.7; 95% *CI*: 2.3, 9.1), hours per day spent on social media (*β* = -1.3; 95% *CI*: -2.3, -0.3), and the number of friends (*β* = 0.3; 95% *CI*: 0.05, 0.5). Finally, resilience was positively associated with life satisfaction and the trust and connections scale (*β* = 1.8; 95% *CI*: 0.9, 2.7 and *β* = 1.8; 95% *CI*: 0.2, 3.4, respectively).

## 1. Introduction

A psychology driven concept of mental resilience refers to a persons' ability to manage everyday life and routine situations, despite the negative conditions they may experience, by either preserving or restoring their mental health in relation to major adversities, such as a potentially traumatic events, difficult circumstances, a pivotal life transition, or a physical disease, [1],[2].

The educational environment is important for the life of students who are going through the late adolescent and post-adolescent phase in order to be able to expand their skills [3]. Necessary conditions in this direction include strengthening their mental resilience, developing their individual confidence, and honing their ability to control their stress levels [4]. Students majoring in nursing and other health-related fields are subject to a variety of study- and career-related pressures; the latter is especially true in the mature stages of health professional training [5]. Stressful interpersonal factors that have been identified to affect their transition to clinical environments include shift work or heavy workloads, as well as feeling inefficiently prepared [6]. Thus, they were more likely to experience mental health issues or burnout symptoms in the course of their studies or later in life [7],[8]. Therefore, these emerging needs demand solid sources of information and should be studied from a young age.

Factors that affect a nursing students' mental resilience have been previously studied, since they often express higher levels of stress or other negative emotions as compared to students from other majors [9],[10]. A study by Devi et al. implied a mediating role of the mental resilience of nursing students to the evolution of stress to later anxiety and depression [11]. A scoping review by Smith et al on the association between resilience and stress in undergraduate nursing students worldwide during COVID-19 pandemic concluded that students with a low resilience reported higher levels of stress [12]. Additionally, cross-sectional studies reported that resilience mediated the relation of academic stress with the quality of life, with resilience having a positive association with the quality of life among nursing students assessed during the pandemic [13],[14].

Even though little is known on the individual and lifestyle characteristics of nursing students that may alter resilience, important factors of related mental health indicators have been identified, such as smoking, alcohol misuse, and impaired physical activity [15]. Participation in sport has shown to positively impact the mental and social health status. Several studies described the impact of sports in the quality of life and the enhancement of resilience to persons with daily actions in sports [16],[17]. The mental resilience of nursing students has been associated with sleep problems and sleep quality [18],[19]. A good quality of sleep has been related to enhanced cognitive function, emotional balance, learning, memory, behavior, quality of life, and mental and physical health [20]. It has been reported that sleep and psychological resilience are two factors that have the potential to alter the relationships between stress and health behaviors [21]. Limited research has been conducted on the association of resilience with body perception or body size in university students, even though body changes are common during the transition from late adolescence to adulthood. However, it is recognized that both these factors affect mental resilience [22]–[25].

Resilience embraces various domains, including tight relationships, stable views, trust in one's instincts, stress lead strengthening and tolerance of negative emotions [26],[27]. Our research hypothesis is that an eventual linkage between resilience and personal promptness to trust [28] might be useful to be identified at the transition and interaction phase of a students' social integration. Trustful feelings and interactions may be theoretically related to the counting of close and secure relationships, where a possible second thought or fear for such “exposure” may be buffered by network support, and all these ingredients might synergistically enhance the ability to adapt and handle negative emotions. The aims of the study are to capture the resilience profile of nursing students and to examine their mental resilience and its association with individual and lifestyle determinants. In addition, a students' mental resilience is investigated in relation to the perceived stress and their tendency to trust those around them in the context of their social connections.

## 2. Materials and methods

### 2.1. Participants

The Ethical Board of the Department of Nursing at the University of Thessaly has the competency as a body to assess and support academic research protocols, research material, app use, information delivery, and participant communication, and involves its academic staff or students to guarantee anonymity and data processing. The consent to use a student institutional email list for research purposes was provided. The study questionnaires were delivered to 250 students' email addresses of all years through an emailing app and the encrypted replies were collected between November 2022 and April 2023. Completion and an email return of the questionnaire were considered equivalent to consent for each participant. The questionnaire was designed to be completely anonymous. No questions sought sensitive personal information, and the responses could not be linked to the individual participants in any way. In total, out of the 250 students that were contacted, 146 students that attended the Department of Nursing at the University of Thessaly (Greece) responded (response rate: 58.4%), which is considered tolerably acceptable for e-surveys.

### 2.2. Ethical approval

The research protocol for the present study was approved by the ethical committee of the Department of Nursing at the University of Thessaly (protocol number 1451/7–10–2022) and complied with ethical standards described in the Declaration of Helsinki.

Informed consent was obtained from all subjects involved in the study.

The data that support the findings of this study are available upon request from the corresponding author.

### 2.3. Individual characteristics and lifestyle habits

Personal information, including sex (male, female), age in years, body mass index (BMI), and family size, were collected. Moreover, lifestyle and everyday habits were recorded, such as smoking, alcohol consumption, hours per day spent browsing the internet, additional hours per day using social media, number of friends, pet ownership, and any sports participation. Additionally, the students were asked if they suffered any health-related problems or chronic diseases during the last year. Finally, a 10-point scale was used to rate life-satisfaction during the last 6 months (from 1 = not at all to 10 = very much).

### 2.4. Psychometric scales

Resilience: In order to quantify the students' resilience, the Greek version of the Connor-Davidson Resilience Scale (CD–RISC–25) was used, which is a consistently reliable tool with a Cronbach's *α* = 0.870. This scale encompasses 25 items, including the following: the willpower to manage challenges, dedication to goals, belief in one's own abilities, viewing change as an opportunity, fostering healthy relationships, adapting to new situations, setting and working towards goals, finding strength in adversity, understanding the value of patience, and effectively handling difficult emotions [26],[27]. The participants responded to each item on a 5-point Likert scale (0 = not true at all, 1 = rarely true, 2=sometimes true, 3 = often true and 4 = true nearly all of the time), which indicated how often the statement applied to them over the past month. The scores ranged from 0 to 100, with higher scores indicating an elevated resilience. The five subscales of CD–RISC–25 are as follows: (1) personal competence (Cronbach's *α* = 0.745); (2) trust in one's instincts (Cronbach's *α* = 0.722); (3) acceptance of change (Cronbach's *α* = 0.618); (4) control (Cronbach's *α* = 0.672); and (5) spiritual influences (Cronbach's *α* = 0.530), as assessed in this context.

Perceived Stress: For the assessment of perceived stress, this study employed the Greek adaptation of the internationally recognized 14-item Perceived Stress Scale (PSS–14) [29],[30]. The PSS–14 equally includes seven positively and negatively worded items (Cronbach's *α* = 0.776), each rated on a 5-point Likert scale ranging from 0 (never) to 4 (very often). The items that were positively phrased assessed the capacity to cope with the perceived stress, whereas the negatively worded items concentrated on evaluating the sense of helplessness, emotional distress, and responses triggered by stressful situations. The highest possible score was 56, which was obtained by adding the scores of the positive questions and the reversed scores of the negative questions [29],[30].

Personal Trust and Connections: In order to measure the students' ability to comprise trustful connections in everyday life, we used the Personal Trust and Connection (PerTC) questionnaire (Cronbach's *α* = 0.701), which consists of 10 items and includes emotional, social, and cognitive reliance meanings [28]. In this context, the scoring was rated on a scale from 1 = Not at all (minimum) to 10 = very much (maximum), and a full-scale scoring was achieved by averaging all ten questions. Through its development, younger age, more years of work experience, dramatic family events, and internet use in free time were all linked with higher total scores. It is a quick and easy scale that is used to assess trustful interaction trends with a good reliability. A reversed scoring for one item was applied [28].

### 2.5. Statistical analysis

A descriptive analysis was performed on the characteristics of the study participants and the distribution of all measured scales. For the analysis of the continuous variables, the mean and standard deviation were calculated; alternatively, frequencies and percentages were implemented for the categorical variables. The Cronbach's alpha coefficient was employed to assess the internal consistency of the scales. To evaluate the bivariate relationships between normally distributed continuous dependent variables and categorical independent variables, either a Student's *t*-test or an analysis of variance (ANOVA) was utilized. The strength of association between the continuous variables following a normal distribution was assessed using Pearson's r correlation coefficient. For variables that were not normally distributed or were discrete, the non-parametric Spearman's r correlation coefficient was employed to evaluate the relationship.

Multivariate linear regression models were employed to assess the relationships between a students' resilience and their personal characteristics, lifestyle and habits, socializing factors, and psychosocial scales, which were found to correlate with CD–RISC–25 in bivariate analyses. We assessed the improvement of the model by entering variables in the regression process in four steps: 1) individual characteristics (i.e., sex and age); 2) life style factors and habits, including smoking, alcohol consumption, night sleep duration, sport participation, preferred relaxing activity, and daily internet use; 3) socializing related factors, such as family size, daily time spent on social media, and the number of friends and peers; and 4) the tools assessing their psychosocial profile, including life satisfaction, stress, and trust. Variables related with *p* < 0.2 at each step were kept within the subsequent model. We evaluated multicollinearity using the variance inflation factor (VIF), with the criterion that if VIF > 10, then this suggests the presence of multicollinearity in the regression model. For all four models, we observed VIF < 2, indicating that multicollinearity did not exist. The expected relationships are shown as *β*-coefficients, along with their corresponding 95% confidence intervals (*CI*). All statistical tests were conducted under the assumption of a two-sided alternative hypothesis and a 5% significance level. The Stata Software, version 13 (Stata Corp LP, College Station, TX, USA), was used for all statistical analyses.

## 3. Results

The study population characteristics are presented in Table 1. The group of 146 students that participated in the study were predominantly female (82.2%), with a mean age of 22 years (*SD* = 6.8). Two out of ten students (20.5%) reported smoking, whereas alcohol consumption was much more common, with 66.4% having at least one drink during a usual week. Almost half of the students (48.0%) participated in sports during the last year. The students' average family size was 4 persons (*SD* = 0.9). Additionally, during the last 6 months, the students reported meeting or being in contact, on average, with at least nine friends and peers (*Mean* = 9.5, SD = 8.3), varying from 0 to 80 people. The daily hours spent on the Internet was 4.2 (*SD* = 1.8) and 2.7 hours/day on social media (*SD* = 1.6). Even though one in three students (37.7%) reported facing a stressful event during the last year, they scored highly on the 1-to-10 life-satisfaction scale (*Mean* = 6.3, *SD* = 1.9), with half of them selecting 7 or above.

A pairwise bivariate correlation of the mental resilience scales, as measured by CD–RISC–25, and the students' perceived stress and their ability to comprise trustful connections, as measured by the PSS-14 and PerTC scales, respectively, are presented in Table 2. Significant positive correlations were observed between the perceived stress and the personal competence and control. Similarly, mental resilience was positively correlated with the students' ability to trust and connect with their peers in the total scales and most of the subscales.

The association of students' personal factors and lifestyle habits with the CD–RISC–25 scale is presented in Table 3. Model 1 included the basic personal characteristics of the participants. Age was found to be significantly associated with resilience (*β* = 0.4; 95% *CI*: 0.1, 0.7) and remained significant after further adjustments.

In Model 2, lifestyle and daily habits were added to the basic model. The resilience scale was found to increase by 1.3 points (95% *CI*: 0.1, 2.4) per hour of night sleep; students that participated in any sport scored higher by 4.2 points (95% *CI*: 0.5, 7.9) compared to those who did not. Finally, resilience was found to decrease by 1.2 points (95% *CI*: -2.2, -0.1) when the hours spent per day on the Internet increased.

**Table 1.** Characteristics of the study population (*n* = 146).

**Project**	***N***	**% or *Mean***	***SD***
**Individual characteristics**			
Age (years)	146	22.0	6.8
Sex			
Male	26	17.8	
Female	120	82.2	
BMI categorization			
Normal (<25 Kg/m^2^)	100	68.5	
Overweight (≥25 Kg/m^2^)	46	31.5	
Chronic illness			
Yes	18	12.3	
No	128	87.7	
**Lifestyle and habits**			
Smoking			
Yes	30	20.5	
No	116	79.5	
Alcohol consumption			
Yes	97	66.4	
No	49	33.6	
Night sleep (hours/day)	146	7.0	1.5
Participation in team sports			
Yes	35	24.0	
No	111	76.0	
Participation in individual sport			
Yes	59	40.4	
No	87	59.6	
Internet use (hours/day)	144	4.2	1.8
Pet ownership			
Yes	86	58.9	
No	60	41.1	
**Socializing**			
Family size (number)	146	4.0	0.9
Social media (hours/day)	144	2.7	1.6
Friends and peers (number)	143	9.5	8.3
**Psychosocial profile**			
Life satisfaction (rating1 to 10)	146	6.3	1.9
Stressful event in the last year			
Yes	55	37.7	
No	91	62.3	
Perceived Stress scale	146	33.0	4.4
Personal Trust and Connections scale	146	6.2	1.1

Note: BMI: Body mass index calculation

**Table 2.** Pairwise correlation of the resilience scales with perceived stress and personal trust and connections.

**Scale**	***Mean***	***SD***	**PSS14**	**PerTC**
Connor-Davidson Resilience-25	64.2	11.8	0.141	0.245**
Personal competence	18.3	4.0	0.198*	0.113
Trust in one's instincts	16.7	4.1	0.004	0.042
Acceptance of change	13.9	2.8	0.048	0.323***
Control	7.6	2.3	0.208*	0.254**
Spiritual influences	5.0	1.9	0.039	0.266**

Note: PSS14: Perceived Stress Scale; PerTC: Personal Trust and Connections. *p < 0.05, **p < 0.01, ***p < 0.001.

**Table 3.** Associations of students' personal factors and lifestyle habits with Connor-Davidson Resilience scale (*n* = 146).

**Project**	**CD–RISC–25**				
	**Model 1** ***β* (95% *CI*)** ***p*-value**	**Model 2** ***β* (95% *CI*)** ***p*-value**	**Model 3** ***β* (95% *CI*)** ***p*-value**	**Model 4** ***β* (95% *CI*)** ***p*-value**	**VIF**
**Individual characteristics**					
Female *vs*. Male	1.3 (-3.7, 6.3) 0.609				1.17
Age (years)	**0.4 (0.1, 0.7)** **0.008**	**0.3 (0.03, 0.6)** **0.033**	**0.5 (0.2, 0.8)** **0.001**	**0.4 (0.1, 0.7)** **0.005**	1.52
**Lifestyle and habits**					
Smoking		3.7 (-1.0, 8.4) 0.126			1.18
Alcohol		3.7 (-0.1, 7.6) 0.059	**4.9 (1.1, 8.7)** **0.011**	1.8 (-2.0, 5.5) 0.359	1.22
Night sleep (hours/day)		**1.3 (0.1, 2.4)** **0.032**	**1.3 (0.2, 2.5)** **0.023**	0.9 (-0.2, 1.9) 0.121	1.11
Any sport participation		**4.2 (0.5, 7.9)** **0.028**	**5.0 (1.3, 8.7)** **0.008**	**5.7 (2.3, 9.1)** **0.001**	1.19
Relaxing activity					
Listen to music		Ref.			
Book, movie, pet		-2.1 (-6.5, 2.2) 0.332			1.41
Dinner/go out with friends		2.4 (-2.1, 6.9) 0.294			1.46
Internet use (hours/day)		**-1.2 (-2.2, -0.1)** **0.028**	-0.4 (-1.6, 0.8) 0.489		1.68
**Socializing**					
Family size			0.7 (-1.4, 2.8) 0.493		1.22
Social media (hours/day)			-1.1 (-2.4, 0.2) 0.092	**-1.3 (-2.3, -0.3)** **0.012**	1.57
Number of friends			**0.3 (0.03, 0.5)** **0.025**	**0.3 (0.05, 0.5)** **0.017**	1.15
**Psychosocial profile**					
Life satisfaction				**1.8 (0.9, 2.7)** **<0.001**	1.25
Perceived stress				0.3 (-0.1, 0.7) 0.190	1.25
Trust and Connections				**1.8 (0.2, 3.4)** **0.028**	1.34

Note: CD–RISC–25: Connor-Davidson Resilience Scale; *CI*: Confidence Intervals; VIF: Variance Inflation Factor. Beta coefficients (*β*) and 95% Confidence Intervals estimated using linear regression models. Model 1 (M1) includes students' personal characteristics; Model 2 (M2) includes factors from M1, plus lifestyle characteristics and habits; Model 3 (M3) includes factors from M2, plus socializing variables; and Model 4 includes factors from M3, plus students' psychosocial profile. Bold fonts indicate statistical significance (*p* < 0.05).

In the next step, socializing factors, such as the number of people that the students interacted with on a daily basis, were added in the previous regression model. The number of friends and peers was associated with increased scores on the CD–RISC–25 scale (β = 0.3; 95% *CI*: 0.03, 0.5). Furthermore, in the final Model 4, a higher life satisfaction and trust were found to be significantly associated with resilience, that is, a point increase in the 1-to-10 life satisfaction scale succeeded to a 1.8-point increase in the CD–RISC–25 scale (95% *CI*: 0.9, 2.7). Similarly, a point increase in the PerTC scale was associated with 1.8-point increase in resilience (95% *CI*: 0.2, 3.4).

## 4. Discussion

We evaluated mental resilience and its association with the individual determinants and lifestyle factors of nursing students in Greece (CD–RISC–25; *Mean* = 64.2, *SD* = 11.8). We collected any pertinent individual feature information to roughly explain the research goals on resilience, perceived stress, and trustful connections. Our results showed strong associations with lifestyle and socializing factors and the students' profile.

Everyday life factors found to associate with a students' resilience were sports participation and night sleep duration. An earlier systematic review suggested that engaging in sports activities was linked to reduced feelings of stress and an enhanced well-being across various aspects, including energy levels, social interactions, psychological state, and overall life contentment [31]. For nursing students that participated in sports, we report a higher score by 4.2 points in the resilience scale. Even though the effect of physical activity may depend on many various factors, such as duration, type, and engagement, there are valuable aspects in sport teamwork, such as the development of interpersonal skills and learning to navigate competition, both in victory and defeat, all of which contribute to the development of resilience [32]; our results enhance this position.

Another finding from the present study was an increase of the resilience scale by 1.3 points per hour of night sleep. Most of the nursing students reported a minimum of 7 hours of sleeping, which complies with the minimum recommended sleep duration of seven hours per night according to the American Academy of Sleep Medicine [33]. Similar results were reported by Duet al. in a multi-centered study from seven countries concerning the length and quality of night sleep from university students during the COVID-19 pandemic [34]. Finally, it has also been associated with various factors such as poor dietary and alcohol misuse, suboptimal academic achievement, an elevated BMI, an increased susceptibility to chronic conditions, and a compromised overall health [35]–[37].

An important factor of impaired resilience is the time spent online and on social media. Previous studies have shown that social media use is associated with mental disorders, including depression and anxiety [38],[39]. In the present study, the total daily time spent on the Internet was 4.2 hours/day and 2.7 hours/day on social media, and both showed a negative effect on resilience. As we move towards consolidation of the concept, a multi-disciplinary analysis and synthesis process is necessary, since a body of heterogeneous research should comply with all potential dimensions [40]. As newly emerged attitudes in the era of the digital “culture” occur, ways to explore the link between stress from various sources among students, Internet use, and the coping ability to prevent related problems deserve attention [17],[41], as mental resilience could be a common denominator in this difficult equation.

Socialization is often overlooked or taken for granted. As human beings, we associate with the multifaceted development of ourselves under the influence of various social factors. In the present study, the number of friends and peers was associated with increased scores of resilience for the nursing students. Results from van Harmelen et al suggest that improving friendship quality or resilient functioning could be beneficial [42]. Previous studies showed that positive, friendly interactions were thought to improve psychological well-being in a variety of ways, including increasing self-esteem, promoting help-seeking and coping behaviors, reducing stress reactivity, and increasing the number of positive interactions [43].

Resilience has been viewed as either an ability, a process, or a psychological trait. We showed that a higher life satisfaction and trust were found to be significantly associated with resilience. Life satisfaction showed a mean of 6.3 [(*SD* = 1.9), range 1–10], perceived stress was assessed as moderate/high with a mean of 33 [(*SD* = 4.4), range 0–56], and trust and connections had a mean of 6.2 [(*SD* = 1.1), range 1–10]. These data approximately depict an average profile of the students being slightly satisfied and trustful in their lives, though with a moderate/high stress perception. These findings pose the need of further research, which requires a purposeful study design, alongside a validated scale on life satisfaction translated into Greek and some qualitative methods to explore how life satisfaction interacts with meanings of trustful connections and resilience. Previous studies indicated that some personal traits were linked with a nursing students' resilience. Indeed, multiple factors have been linked to an enhanced resilience, notably including a high self-efficacy [44],[45].

By focusing on eight studies that linked students who delivered social support to increased resilience levels, a review by Aryuwat et al highlighted the importance of social support for a nursing students' resilience [46].

The present cross-sectional study has several limitations. This research is limited to only one nursing department of University of Thessaly; thus, the findings cannot be generalized. Additionally, the data came from a non-random, small number of participants. A mixed methodological approach is also needed to detect how body perception or size is related with a students' everyday behaving. BMI may offer some useful information. However, weight and height were self-reported and did not necessarily describe their personal beliefs on their physical appearance. We acknowledge some related limitations for both reasons.

However, the present study provides valuable information to the educators, who could implement intervention programs in nursing schools in order to strengthen resilience and to reduce stress levels or burnout later in life [47]. Cultivating a students' resilience can potentially nurture a culture of resilience and well-being among nurses [48]. We believe that students of the late generations may have a different conception of their everyday interactions, through digital communication, many e-contacts, shorter verbal discussions, and perhaps a different way to handle unpleasant or difficult routine situations. In that manner, even meanings of trust and its expressions may be vague for researchers. We found that sport participation, having multiple friends, and having a simple self-reported overall assessment of life satisfaction and trust are associated with an enhanced resilience, while social media use shows a reversed trend. This information is important for any reform which aspires to improve educational strengthening and any public health initiative to collect local data from academic communities. In a changing and unsolid global environment, it is important to prepare future citizens by offering as much as we can from family and societal resources and by respecting individual diversity. A balanced mix of the above ingredients may be necessary to buffer weaknesses and to enhance individual and collective potentials.

## 5. Conclusion

We presented data on the resilience of students from the Department of Nursing at the University of Thessaly, Greece. Our data evaluated mental resilience and captured its correlations with individual determinants and their living conditions. Although a minority of the nursing students faced a stressful event during the last year, the majority of them encouraging scored highly on the life-satisfaction scale. In addition, most of them reported at minimum of 7 hours of sleeping, which is rationally associated with an increase in the resilience scale. Furthermore, a negative correlation was found between the time that nursing students spent on the internet and on social media as a daily routine with their mental resilience to cope with stressful situations. On the other hand, participation in sports was shown to positively impact their mental and social status, which was verified in our study, since the nursing students who participated in sport scored highly in the resilience scale. Finally, in our socialization study, in terms of the number of friends, life satisfaction and trust was connected with increased scores of resilience, which identified the essential role of social support and connections. Further research needs to be conducted on how to consecutively collect data on resilience of health profession students in order to expand observations based on a larger number of participants from different settings by drawing more robust and validated findings.

## Use of AI tools declaration

The authors declare they have not used Artificial Intelligence (AI) tools in the creation of this article.
